# Quality Control of Photosystem II: Lipid Peroxidation Accelerates Photoinhibition under Excessive Illumination

**DOI:** 10.1371/journal.pone.0052100

**Published:** 2012-12-27

**Authors:** Tiffanie Chan, Yurika Shimizu, Pavel Pospíšil, Nobuyoshi Nijo, Anna Fujiwara, Yoshito Taninaka, Tomomi Ishikawa, Haruka Hori, Daisuke Nanba, Aya Imai, Noriko Morita, Miho Yoshioka-Nishimura, Yohei Izumi, Yoko Yamamoto, Hideki Kobayashi, Naoki Mizusawa, Hajime Wada, Yasusi Yamamoto

**Affiliations:** 1 Graduate School of Natural Science and Technology, Okayama University, Okayama, Japan; 2 Department of Biophysics, Centre of the Region Haná for Biotechnological and Agricultural Research, Faculty of Science, Palacký University, Olomouc, Czech Republic; 3 Institute of Plant Science and Resources, Okayama University, Kurashiki, Japan; 4 Center for Faculty Development, Okayama University, Okayama, Japan; 5 Department of Life Sciences, Graduate School of Arts and Sciences, University of Tokyo, Tokyo, Japan; University of Hyderabad, India

## Abstract

Environmental stresses lower the efficiency of photosynthesis and sometimes cause irreversible damage to plant functions. When spinach thylakoids and Photosystem II membranes were illuminated with excessive visible light (100–1,000 µmol photons m^−1^ s^−1^) for 10 min at either 20°C or 30°C, the optimum quantum yield of Photosystem II decreased as the light intensity and temperature increased. Reactive oxygen species and endogenous cationic radicals produced through a photochemical reaction at and/or near the reaction center have been implicated in the damage to the D1 protein. Here we present evidence that lipid peroxidation induced by the illumination is involved in the damage to the D1 protein and the subunits of the light-harvesting complex of Photosystem II. This is reasoned from the results that considerable lipid peroxidation occurred in the thylakoids in the light, and that lipoxygenase externally added in the dark induced inhibition of Photosystem II activity in the thylakoids, production of singlet oxygen, which was monitored by electron paramagnetic resonance spin trapping, and damage to the D1 protein, in parallel with lipid peroxidation. Modification of the subunits of the light-harvesting complex of Photosystem II by malondialdehyde as well as oxidation of the subunits was also observed. We suggest that mainly singlet oxygen formed through lipid peroxidation under light stress participates in damaging the Photosystem II subunits.

## Introduction

Although light is needed for photosynthetic energy conversion, excessive light can be harmful to plants. Photoinhibition is a phenomenon in which photosynthesis is inhibited under excessive illumination [1]. Photosystem II (PSII) is the primary target of photoinhibition, and the reaction center binding D1 protein is extremely vulnerable to attack by endogenous cationic radicals and reactive oxygen species (ROS) produced under excessive illumination [2], [3], [4]. It is well known that singlet oxygen (^1^O_2_), one of the ROS, is generated by strong illumination of PSII through interaction of the excited triplet state of P680 (the primary electron donor of PSII) and molecular oxygen [5], [6], [7], [8], [9], [10], [11]. Under strong illumination conditions, oxygen is also reduced by the primary electron acceptor pheophytin and plastoquinone (PQ) molecules, which results in the formation of superoxide anion radicals [12], [13]. Recently, hydroxyl radicals (HO^•^) were shown to be produced on the acceptor side of PSII by high light [14], [15]. Damage to PSII by ^1^O_2_ and HO^•^ produced under these light stress conditions explains the so-called “acceptor-side photoinhibition” of PSII [1], [2], [3], [4], [16]. When PSII is illuminated after the donor side is inactivated, endogenous cationic radicals such as P680^+^, the oxidized form of the primary electron donor of PSII, and Tyr_Z_^+^, the oxidized form of the secondary electron donor of PSII, are produced because of poor donation of electrons from a water-splitting 4Mn-Ca complex at the reaction center. These radicals subsequently cause damage to the D1 protein. This process is induced by relatively weak light and is referred to as the “donor-side photoinhibition” of PSII [17], [18]. The damage by the donor-side photoinhibition is confined to the donor-side of PSII, i.e. the luminal side of the D1 protein.

Because the PSII complexes are embedded in the lipid bilayer in the thylakoid membrane, they are inevitably affected by the conditions of lipids under stress conditions. Lipids with polyunsaturated fatty acids containing two or more double bonds are potential targets of peroxidation [19], [20]. The lipid peroxidation process consists of the so-called “initiation”, “propagation” and “termination” steps. Typically, HO^•^ initiates peroxidation of fatty acids by abstracting a hydrogen atom from an active methylene group where the HO^•^ becomes H_2_O. The resulting lipid alkyl radical (L^•^) reacts with a molecular oxygen to form lipid peroxyl radical (LOO^•^). The LOO^•^ then abstracts a hydrogen atom from an adjacent fatty acid side chain to become a lipid peroxide (LOOH), and this step induces propagation in lipid peroxidation.Recently, it was shown that lipid peroxidation takes place when spinach thylakoids and PSII membranes are subjected to moderate heat stress at 40°C for 30 min [21]. It was also demonstrated that ^1^O_2_ and HO^•^ are generated in spinach PSII membranes exposed to moderate heat stress, which likely takes place at both the acceptor and donor sides of PSII [21], [22]. Degradation of the D1 protein, release of Mn and liberation of PsbO, P and Q proteins from PSII are all suggested to occur with the heat-induced lipid peroxidation [21].

In the present study, we investigated possible participation of lipid peroxidation in photodamage and modification of PSII under photoinhibitory illumination using spinach thylakoids and PSII membranes. Our study, in which the effects of externally added lipoxygenase (LOX) in the dark and those of illumination with excessive light were compared, suggests that the lipid peroxidation under the illumination of the membrane samples causes damage to PSII, in particular to the D1 protein and the light-harvesting chlorophyll-proteins of PSII (LHCII), and modification of the latter components by malondialdehyde (MDA) as well.

## Results

### Inhibition of PSII Activity by Illumination of the Thylakoids and PSII Membranes

**Figure 1:**
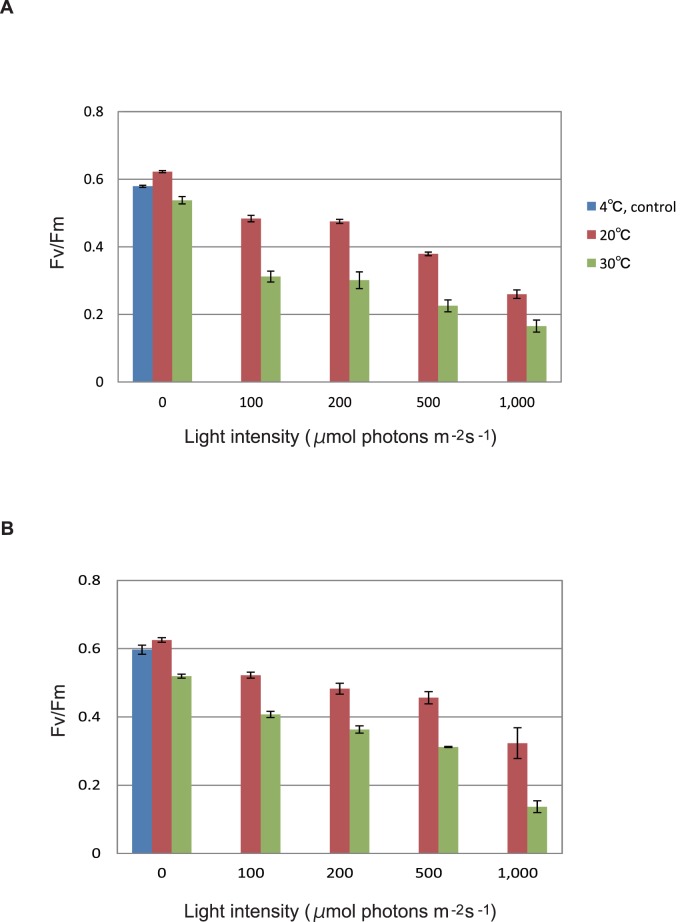
Light-induced decrease in the PS II activity of spinach thylakoids and PS II membranes monitored by chlorophyll fluorescence Fv/Fm. (**A**) The thylakoids. (**B**) The PSII membranes. The samples were incubated in solution B and kept in the dark or illuminated with white light with given light intensities (100–1,000 µmol photons m^−2^ s^−1^) at either 20°C (brown bars) or 30°C (green bars) for 10 min. Dark control kept at 4°C is also shown (blue bars). After incubation in the dark for 30 min on ice, chlorophyll fluorescence was measured with a Mini-PAM at 25°C. The data are the means of three measurements±S.D.

We first examined the effects of illumination with white light of various light intensities on PSII activity of spinach thylakoids (Fig. 1A) and PSII membranes (Fig. 1B), by monitoring the *quantum* efficiency of PS II photochemistry determined as the ratio of the variable to maximal chlorophyll fluorescence (Fv/Fm). The Fv/Fm values of the dark control samples were around 6, and the relatively low Fv/Fm values compared with those of starting materials (spinach leaves in which Fv/Fm was around 8) were probably due to damage to PS II through lipid peroxidation during the sample preparation. In the present study we omitted Na-ascorbate in the grinding medium of spinach leaves to avoid the effect of the reductant on lipid peroxidation that we measured subsequently under light stress (see Materials and Methods). The membrane samples were illuminated at 100–1,000 µmol photons m^−2^ s^−1^ for 10 min at either 20°C or 30°C. After the illumination, the PSII activity decreased both in the thylakoids and PSII membranes depending on the light intensity, and the loss of the activity was more significant at 30°C compared with 20°C. At 30°C, even dark incubation of the samples for 10 min caused a decrease in Fv/Fm, probably reflecting increase in lipid peroxidation at higher temperatures.

### Lipid Peroxidation Caused by Illumination or by Addition of Lipoxygenase to the Thylakoids and PSII Membranes

**Figure 2:**
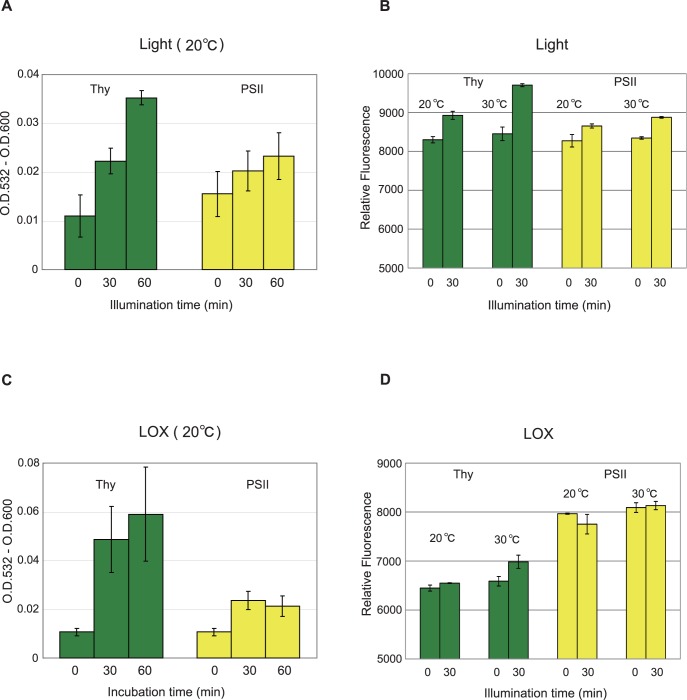
Lipid peroxidation induced by illumination or by the addition of soybean lipoxygenase to the thylakoids and PS II membranes. (**A**) Lipid peroxidation in the thylakoids (green bars) and PSII membranes (yellow bars) illuminated with high light (intensity, 1,000 µmol photons m^−2^ s^−1^) at 20°C for indicated periods. Lipid peroxidation was assayed by the TBARS assay. (**B**) Lipid peroxidation assayed using a fluorescence probe Spy-LHP in the thylakoids (green bars) and PSII membranes (yellow bars) illuminated either at 20°C or 30°C for 30 min with high light (the intensity was the same as that used in (**A**). Dark controls at 20°C and 30°C are also shown. (**C**) Lipid peroxidation in the thylakoids (green bars) and PSII membranes (yellow bars) induced by incubation with lipoxygenase (LOX, 0.1 mg mL^−1^) for indicated periods. The TBARS assay was used for lipid peroxidation assay. (**D**) Lipid peroxidation in the thylakoids (green bars) and PSII membranes (yellow bars) induced by incubation with LOX at either 20°C or 30°C for 30 min. The concentration of LOX was 0.1 mg mL^−1^. Spy-LHP was used for lipid peroxidation assay. The data are the means of three measurements±S. D.

We measured lipid peroxidation using the thiobarbitulic acid (TBA) reactive substance (TBARS) method, which has been generally used for the assay of lipid peroxidation (Fig. 2A). Illumination of the thylakoids and PSII membranes with strong light (1,000 µmol photons m^−2^ s^−1^) for 30 min induced lipid peroxidation, and the level of peroxidation was higher in the thylakoids than in the PSII membranes.

Strong light-induced lipid peroxidation was also measured using fluorescence probe 2-(4-diphenylphosphanyl-phenyl)-9-(1-hexyl-heptyl)-anthra[1,1,9-def,6,5,10-d′e′f′]diisoquinoline-1,3,8,10-tetraone (Spy-LHP) (Fig. 2B). Spy-LHP reacts with lipid peroxide specifically and was successfully applied to monitor lipid peroxidation in spinach PSII membranes in a previous study [23]. When the samples were illuminated (light intensity, 1,000 µmol photons m^−2^ s^−1^) at 20°C for 30 min, lipid peroxidation took place, and we noted again that the extent of the excessive light-induced lipid peroxidation was larger in the thylakoids compared with the PS II membranes.

We next examined lipid peroxidation by adding soybean LOX (final concentration, 0.1 mg mL^−1^) to the samples at either 20°C or 30°C in the dark with the TBARS method (Fig. 2C) and the fluorescence measurement using Spy-LHP (Fig. 2D). LOX is an iron-containing enzyme widely found in plants, fungi and animals, which catalyzes oxidation of lipids to lipid peroxides [24], [25]. The substrates of the enzymes are polyunsaturated fatty acids containing *cis* double bonds. We observed LOX-induced lipid peroxidation both in the thylakoids and the PSII membranes by the TBARS method, and the extent of lipid peroxidation by LOX was larger in the thylakoids than in the PSII membranes (Fig. 2C). When we used Spy-LHP, LOX-induced lipid peroxidation was detected in the thylakoids, but the lipid peroxidation was not so clear in the PSII membranes (Fig. 2D).

In spinach thylakoids, an endogenous LOX, which shows homology to soybean LOX, was not detected by Western blot analysis using a specific antibody against soybean LOX (Fig. S1). Thus, the lipid peroxidation observed here under illumination should not have resulted from activation of the endogenous LOX but from the other processes specific to the illumination, most probably ROS-dependent processes.

### Detection of Malondialdehyde-protein Adducts in the Thylakoids Under Strong Illumination or by Incubation with Lipoxygenase in the Dark

**Figure 3:**
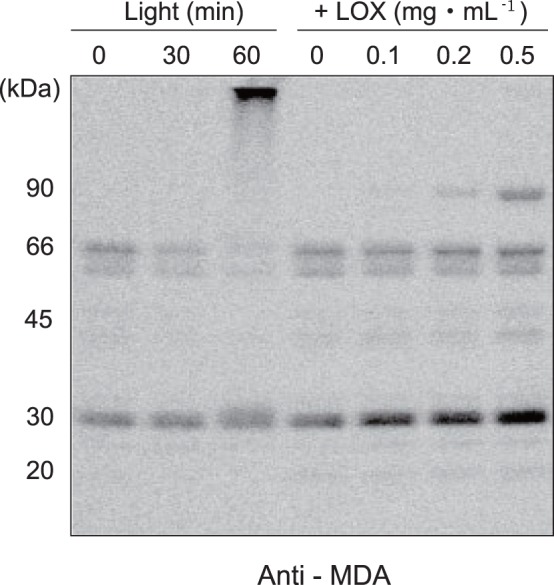
Formation of malondialdehyde-protein adducts in the thylakoids under light stress or in the presence of lipoxygenase. Spinach thylakoids were either illuminated with strong illumination (1,000 µmol photons m^−2^ s^−1^ for 30 and 60 min, denoted as “Light” at the top of the gel), or treated with LOX (0.1–0.5 mg mL^−1^, denoted as “+LOX”), and the production of malondialdehyde (MDA)–protein adducts was examined by Western blot analysis with an anti-MDA monoclonal antibody (Nichiyu, Japan). The molecular markers are shown at the left side of the fluorogram.

Through lipid peroxidation, malondialdehyde (MDA: molecular mass, 72.1) is produced as a secondary product. MDA is known to form adducts with proteins and nucleic acids [26]. We detected MDA-protein adducts in the thylakoids illuminated with excessive light by Western blot analysis with a specific antibody against MDA (Fig. 3). The thylakoids contained a low level of MDA-protein adducts having molecular masses of 60–66 kDa even when they were incubated in the dark without further treatment. As we have already suggested in Fig. 1, this may be due to peroxidation of the lipids in the samples during the preparation steps. When the thylakoids were illuminated with strong light (1,000 µmol photons m^−2^ s^−1^) at 20°C, the amounts of the 60–66 kDa adducts decreased, and instead, new bands appeared at the higher molecular mass range. This is probably ascribed to either MDA-mediated aggregation of the proteins or cross-linking of MDA to the aggregated proteins produced under strong illumination.

These results also support the results obtained in Fig. 2, indicating that strong light induces lipid peroxidation in the thylakoids and with less extent in the PSII membranes. Production of the MDA-protein adducts was also observed with thylakoids when LOX (0.1–0.5 mg ml^−1^) was added. In this case, larger protein adducts associated with MDA were also detected. Among several MDA-protein adducts found in the fluorogram, the major adducts have a relative molecular mass of about 30,000 Da, suggesting that these products are probably formed by addition of MDA to LHCII. Modification of the D1 protein by MDA may be eliminated by the reason described later in Discussion.

### Damage to the Proteins of PSII by Strong Illumination

It is well known that excessive illumination of PSII induces degradation of the D1 protein. Aggregation of the photodamaged D1 protein with the D2 protein, CP43 and the α-subunit of cytochrome *b*_559_ has also been reported [27], [28]. The degradation and aggregation of the D1 protein are a good measure of photodamage to the protein [3]. Here, we found in the thylakoids that in addition to the D1 protein, the subunits of LHCII are also susceptible to photodamage (Fig. S2). A Coomassie blue-stained gel of SDS/urea-PAGE and the subsequent Western blot analysis using antibodies against the LHCII subunits show that the amount of LHCII is decreased by excessive illumination (Fig. S2A and B). The effect of excessive light was more prominent in the thylakoids than in the PSII membranes. The loss of the LHCII band is partly due to aggregation of LHCII subunits with the D1 protein (Fig. S2C). We also observed loss of CP43 under strong illumination and the accompanying aggregation of the D1 protein and CP43, which was reported previously [29].

### Inhibition of Photosystem II Activity and Damage to the D1 Protein and LHCII by Incubation of the Thylakoids and PSII Membranes with Lipoxygenase in the Dark

**Figure 4:**
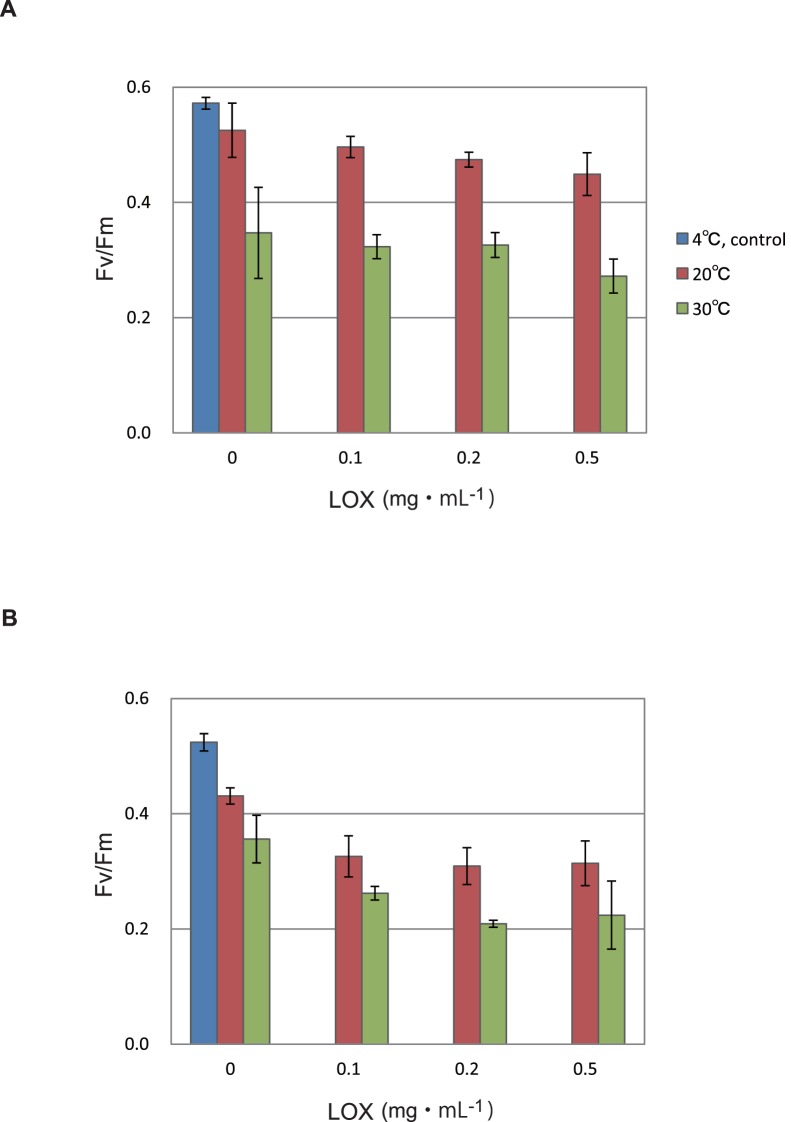
Effects of lipoxygenase on PSII activity in the thylakoids and PSII membranes. To monitor the PSII activity, chlorophyll fluorescence Fv/Fm was measured. (**A**) The thylakoids. (**B**) The PSII membranes. LOX was added at the concentration of 0.1, 0.2 and 0.5 mg mL^−1^, and the reaction mixtures were incubated at either 20°C (brown bars) or 30°C (green bars) for 30 min. Controls incubated at 4°C without LOX are also shown (blue bars). The data are the means of three measurements±S. D.

When we treated the thylakoids and PSII membranes with 0.1 mg mL^−1^ LOX, the PSII activity measured by chlorophyll fluorescence Fv/Fm decreased by ∼20% in the thylakoids and PSII membranes after 30 min incubation at 20°C in the dark (Fig. 4). The loss of the PSII activity increased when the samples were treated with LOX at 30°C.

**Figure 5:**
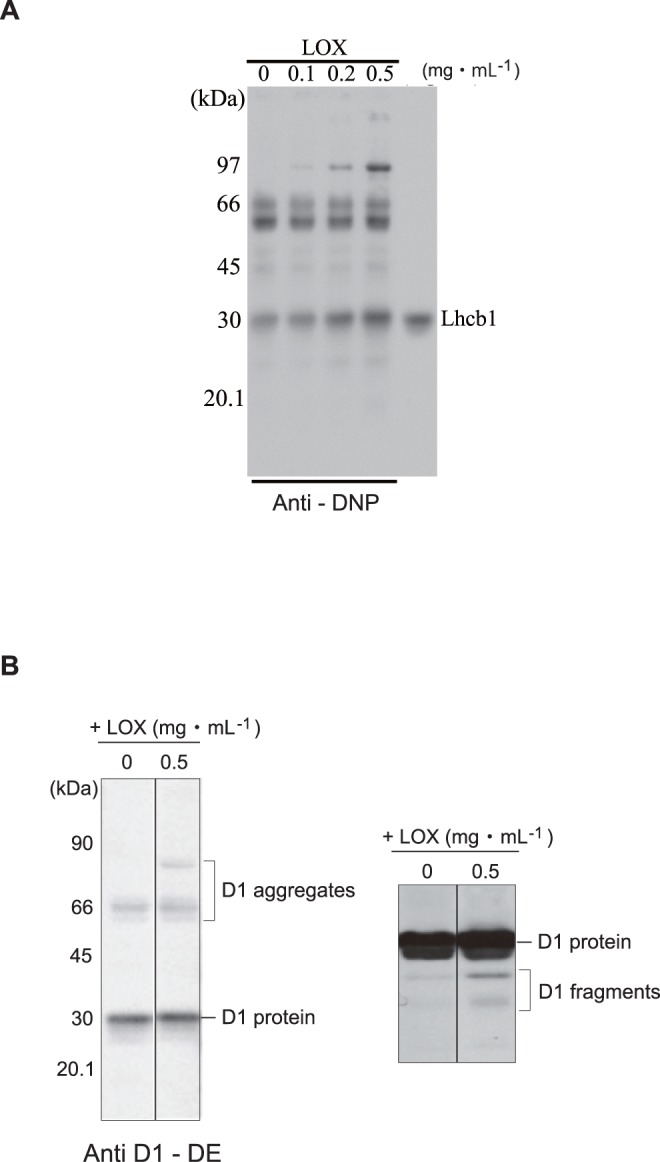
Oxidation and subsequent aggregation and degradation of the LHCII and the D1 protein in the thylakoids by the addition of lipoxygenase (LOX). (**A**) A fluorogram of Oxi-Blot analysis showing oxidation, aggregation and degradation of the proteins in the thylakoids by the addition of LOX. The concentration of LOX is 0.1, 0.2 and 0.5 mg mL^−1^. Other conditions were the same as those in Fig. 3. The position of Lhcb1 is indicated at the fluorogram on the right side. (**B**) Fluorograms showing aggregation of the D1 protein (left) and degradation of the D1 protein (right) by the addition of 0.5 mg mL^−1^ LOX. Western blot analysis was done using an antibody against the DE-loop of the D1 protein. Molecular markers and the positions of the D1 protein, D1 aggregates, and D1 degradation products are shown on the left side and right side of the fluorograms, respectively. The degradation products of the D1 protein by the addition of LOX was detected by overexposure of the gel (right).

Since the addition of LOX to the thylakoids and PSII membranes induced inhibition of PSII activity of the sample, we next examined whether the LOX added to the thylakoids induces oxidation of the D1 protein and LHCII in the dark and damages these proteins. We measured oxidation of the proteins using an Oxi-Blot protein oxidation detection kit (Chemicon International, USA), and estimated the carbonyl content of the proteins by Western blot analysis with an antibody against 2,4-dinitrophenyl (DNP) (see Materials and Methods). In the control thylakoids without addition of LOX, several proteins including Lhcb1, a subunit of the light-harvesting chlorophyll-protein complex which has a molecular mass of ∼ 30 kDa, and other proteins with higher molecular masses were already oxidized. Incubation of the thylakoids with increasing concentrations of LOX at 20°C further oxidizedLhcb1 and induced formation of protein aggregates at higher molecular mass ranges (Fig. 5A). Importantly, the externally added LOX also caused damage to the D1 protein, which was manifested in the aggregation (Fig. 5B, left) and degradation of the D1 protein (Fig. 5B, right) in the thylakoids.

### Effects of Malondialdehyde on the PSII Activity

As described above, MDA is one of the aldehydes produced through lipid peroxidation. We examined the effects of externally added MDA on PSII activity of the thylakoids (Fig. S3). The thylakoids incubated with100 µM MDA at 30°C for 30 min showed decrease in Fv/Fm by ∼20%, while the loss of the activity was smaller at 20°C.

### Detection of ^1^O_2_ in the PSII Membranes Induced by Strong Illumination and by Addition of Lipoxygenase with EPR Spin Trapping

**Figure 6:**
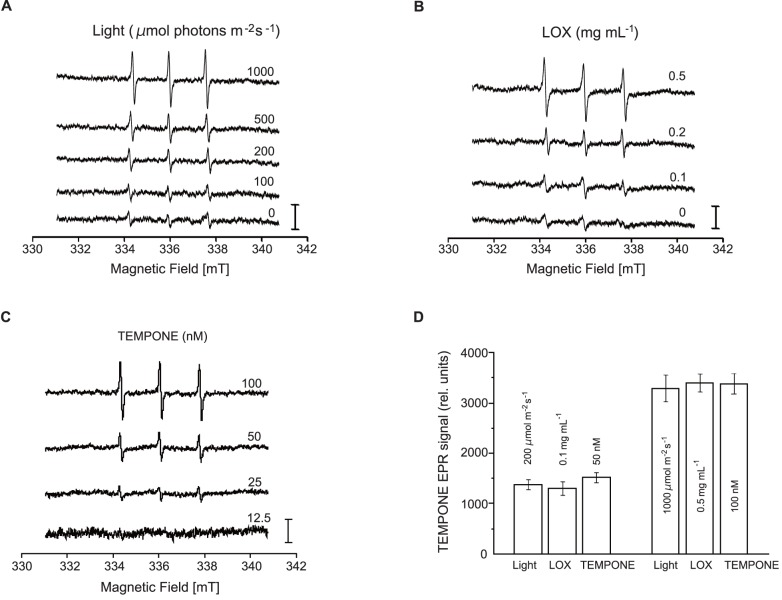
Production of ^1^O_2_ in PSII membranes by strong illumination and addition of lipoxygenase. (**A**) TEMPONE EPR spectra were measured in PSII membranes exposed to strong illumination (intensity, 100–1,000 µmol photons m^−2^ s^−1^, illumination time 30 min). The illumination was performed in the presence of 50 mM TMPD, 500 µg chlorophyll mL^–1^ and 40 mM MES (pH 6.5). (**B**) TEMPONE EPR spectra were measured in PSII membranes treated with LOX (0.1, 0.2 and 0.5 mg mL^−1^). The LOX treatment was performed in the presence of 50 mM TMPD, 500 µg chlorophyll mL^–1^ and 25 mM phosphate buffer (pH 7). (**C**) TEMPONE EPR spectra measured in pure TEMPONE dissolved in DMSO. (**D**) Relative intensity of TEMPONE EPR spectra obtained after weak light (200 µmol photons m^−2^ s^−1^), LOX (0.1 mg mL^–1^), 50 nM TEMPONE and strong light (1,000 µmol photons m^−2^ s^−1^), LOX (0.5 mg mL^–1^), 100 nM TEMPONE. Signal intensity was evaluated as a relative height of the central peak. The vertical bars represent 1,200 relative units. The data are the means of three measurements±S. D.

It was shown previously that strong illumination of the PSII membrane causes production of ^1^O_2_ [5], [8]. These results were confirmed in the present study by an EPR spin-trapping technique employing the hydrophilic spin trap compound 2, 2, 6, 6-tetramethyl-4-piperidone (TMPD) (Fig. 6). The addition of TMPD to PSII membranes in the dark did not result in the appearance of a 2, 2, 6, 6-tetramethyl-4-piperidone-1-oxyl (TEMPONE) EPR signal (Fig. 6A). The negligible TEMPONE EPR signal observed in non-illuminated PSII membranes was due to impurities in the spin trap. The exposure of PSII membranes to light (100–1,000 µmol photons m^−2^ s^−1^ for 30 min) in the presence of TMPD resulted in the generation of a TEMPONE EPR signal (Fig. 6A). These observations confirm that the exposure of PSII membranes to strong light leads to the production of ^1^O_2_. To explore whether lipid peroxidation leads to ^1^O_2_ production, TEMPONE EPR spectra were measured in PSII membranes treated with LOX. When PSII membranes were treated with LOX at 30°C in the presence of TMPD, a TEMPONE EPR signal was observed (Fig. 6B). These results indicate that the initiation of lipid peroxidation by exogenous LOX leads to the production of ^1^O_2_. To quantify the number of spins trapped by TEMPD in the polar phase, TEMPONE EPR signals observed after illumination and LOX treatments were compared to TEMPONE EPR signal obtained using pure TEMPONE (Fig. 6C). The number of spins trapped in the polar phase after weak light (200 µmol photons m^−2^ s^−1^) and LOX (0.1 mg mL^–1^) treatments corresponds to the number of trapped spins in the range of several tens of nM. When strong light (1,000 µmol photons m^−2^ s^−1^) and LOX (0.5 mg mL^–1^) was used, the number of spins trapped by TEMPD in the polar phase ranged in the hundreds of nM (Fig. 6D).

## Discussion

Lipids play fundamental roles in the primary reactions in photosynthesis because they are the major constituents of the thylakoid membranes [30], [31]. The most abundant lipids in the thylakoids from higher plants are two galactolipids, monogalactosyldiacylglycerol (MGDG) and digalactosyldiacylglycerol (DGDG), which constitute 50–60 mol% and 20–30 mol% of the total lipids, respectively [32]. There are also phospholipid, phosphatidylglycerol (PG), and sulfolipid, sulfoquinovosyldiacylglycerol (SQDG). PG and SQDG comprise 5–10 mol% of the total lipids [33]. Highly unsaturated fatty acids (18∶3 and 16∶3) account for ∼70 mol% of fatty acids in thylakoids and >90 mol% of the fatty acids in MGDG [32]. In spinach, which we used in the present study, PG was shown to comprise 12.6 mol% and 25.3 mol% of total lipids in the thylakoids and PSII complexes, respectively, being the only phospholipid in these samples [34].

One of the outstanding features of lipids in the PSII complex revealed by a recent X-ray crystallographic analysis of the PSII complex from the cyanobacterium *Thermosynechococcus elongatus* [35] is that the distribution of lipid is not random but is functionally fixed and each lipid molecule surrounding the helical transmembrane proteins in PSII seems to have a specific role [35], [36]. The interface between lipids and the acceptor side of the D1 protein where Q_B_ is located plays a significant role as one of the most important rate-limiting steps in the overall electron transport of PSII. The Q_B_ site is the site where the secondary PQ electron accepter is located, and from the 2.9-Å resolution data of the X-ray crystallography of the PSII complex, several lipids that form the PQ diffusion cavity have been identified [36]. They are PG, MGDG, DGDG, and SQDG [35], [36], [37]. The fatty acids of the lipids comprising the PQ diffusion cavity should be polyunsaturated and maintain the fluidity of the lipid phase to enable efficient migration of the PQ molecules that are bound to and released from the Q_B_ site for replacement. Membrane fluidity is also important for efficient turnover of the D1 protein [38]. Thus, the fatty acids in the lipids surrounding the D1 protein are most likely polyunsaturated in the native thylakoid membranes. Polyunsaturated fatty acids are, however, inevitably peroxidized under oxidative conditions because of the presence of double bonds in the acyl groups [39].

In the present study, we showed that lipid peroxidation takes place upon the illumination of spinach thylakoids and PSII membranes with visible light (1,000 µmol photons m^−2^ s^−1^) and the level of lipid peroxidation was higher at 30°C than at 20°C (Fig. 2). We employed two methods to measure lipid peroxidation: the TBARS assay and the fluorometric method using a fluorescent probe Spy-HLP. The TBARS assay depends on the reaction of TBA with malondialdehyde (MDA) that is one of the secondary products of lipid peroxidation. The biggest disadvantage of the method is that the absorbance at 532 nm due to the TBA-MDA adduct is affected by some sugars such as sucrose and pigments such as anthocyanin if they are in the reaction mixture. In our measurement, the effects of the interfering substances were checked carefully beforehand. Another shortcoming is that MDA tends to bind proteins and nucleic acids and therefor it is not easy to estimate the total amount of MDA. The assay kit we used here (a NWLSS malondialdehyde assay kit, Northwest Life Science, USA) was developed to minimize the problem, according the manufacture’s protocol. Spy-LHP reacts with lipid peroxides LOOH specifically. However, since LOOH is usually unstable, we may not able to estimate the real amount of LOOH and may underestimate it under certain conditions. This may be the reason why we detected a smaller lipid peroxidation when we used Spy-LHP, compared with the TBARS method. Thus we had to use the two methods simultaneously to estimate the lipid peroxidation.

Lipid peroxidation of thylakoids is induced not only by strong illumination but also by heat stress. From earlier work, it was shown that when thylakoids are exposed to high temperatures such as 70–90°C and 120–140°C, a chemiluminescence signal appeared, which indicates production of ^1^O_2_ and triplet carbonyls during heat-induced lipid peroxidation and/or thermolysis of lipid hydroperoxides [40]. However, we recently showed that lipid peroxidation occurs in spinach thylakoids and PSII membranes not at such high temperatures but at a more moderate high temperature: when these membrane samples were incubated at 40°C for 30 min, prominent peroxidation of lipids took place [21]. Under the mild heat stress, we also detected production of ^1^O_2_ and HO^•^ in the PSII membranes.

The mechanism of initiation in the lipid peroxidation under moderate heat stress is not absolutely clear. Conceivably highly oxidizing species such as ferryl and perferryl could be involved in this process [21]. Non-heme iron, heme iron and even the Mn atoms released from the water splitting center of PSII in the reduced form may be able to contribute to lipid peroxidation because generally transition metals can initiate fatty acid peroxidation by reaction with molecular oxygen with the subsequent generation of ^1^O_2_ and HO^•^ [20]. It is possible that heat stress facilitates release of these transition metals from their functional sites.

By contrast, it is rather easy for us to assume the factors necessary to trigger lipid peroxidation under light stress. Probably HO^•^ formed by photochemical reaction of PSII at either the acceptor side or donor side of PSII under strong illumination [14], [15], [41] can initiate the lipid peroxidation. In the photoinhibition of PSII, production of ^1^O_2_ near the reaction center under excessive illumination and the damaging effect of ^1^O_2_ to PSII have been well documented. Thus, we should consider the possibility that ^1^O_2_ produced by the photochemical reaction participates in lipid peroxidation near PSII. It was shown that unsaturated fatty acids react readily with ^1^O_2_, and this reaction proceeds through a different mechanism than the HO^•^-triggered lipid peroxidation [20]. Namely, singlet oxygen molecules react directly with double bonds of fatty acids to yield allylic hydroperoxides, LOOH. The lifetime of ^1^O_2_ is known to be longer in the interior of membranes than in an aqueous environment, and hence it is highly possible that the reaction between ^1^O_2_ and unsaturated fatty acids of lipids takes place inside the thylakoid membranes during the strong illumination.

Lipid peroxidation took place when the thylakoids were incubated with LOX (Fig. 2). LOXs contain non-heme irons [iron (III)], which abstract hydrogen atoms from allylic C-H bonds in fatty acids [42]. Like the non-enzymatic lipid peroxidation, the resulting lipid alkyl radical (L^•^) reacts with molecular oxygen to form lipid peroxyl radical (LOO^•^), which finally forms lipid peroxide (LOOH) through the subsequent reaction with H abstracted from an adjacent unsaturated fatty acid. The level of lipid peroxidation induced by LOX was not so high (Fig. 2). Probably the amount of LOX added to the samples in the present study was enough to attain the maximum lipid peroxidation if it works properly. The small activity of lipid peroxidation observed with the externally added LOX is probably due to poor accessibility of the LOX enzyme to the substrate fatty acids in the membrane lipids.

In the thylakoids and PSII membranes, bound lipids may surround the PSII complexes. Because these bound lipids may be deeply embedded in the crevices of proteins and protein complexes, LOX may not able to reach the lipids and the constituent fatty acids easily, unless the fatty acids are released from the lipid bilayers. More importantly, LOX was less effective in the PSII membranes than in the thylakoids. These results indicate that the lipids in the PSII complexes are more resistant to lipid peroxidation. The proportion of bound lipid to free lipid in the PSII membranes should be higher than that in the thylakoids, and therefore it is reasonable to assume that tolerance to lipid peroxidation in the PSII membranes is attributable to the abundance of bound lipids.

Strong illumination or addition of LOX in the dark to the thylakoids induced oxidation of some proteins in PSII. Formation of cross-linked products between MDA and the LHCII subunits (Fig. 3) suggests that lipid peroxidation takes place near these proteins and causes damage to the proteins. MDA is a water-soluble substance, having a long life and therefore being able to move over a long range on the thylakoids to the targets. Thus, MDA may react with various membrane proteins once it is generated after lipid peroxidation. However, MDA reacts in particular with the lysine residues of proteins, and because spinach D1 protein contains no lysine, it is unlikely that MDA reacts with and chemically modify the D1 protein.

Importantly, we detected ^1^O_2_ by EPR spin trapping through lipid peroxidation by incubating the PSII membranes with LOX (Fig. 6). ^1^O_2_ is also produced when the PSII membranes are illuminated by excessive light [1], [2], [3], [4]. It is well known that ^1^O_2_ is responsible for the damage to the D1 protein in the acceptor side photoinhibition of PSII. Thus, ^1^O_2_ produced through lipid peroxidation probably damages the D1 protein and the LHCII subunits as well.

We detected aggregation and degradation of the D1 protein in the dark by the addition of LOX to the thylakoids (Fig. 5). These data suggest that the D1 protein is damaged through lipid peroxidation. We compared the pattern of degradation/aggregation of the D1 protein induced by the addition of LOX with that induced by strong light, and found that the both are very similar to each other. The damage to LHCII was another prominent effect of strong light to PSII (Fig. S2).

**Figure 7:**
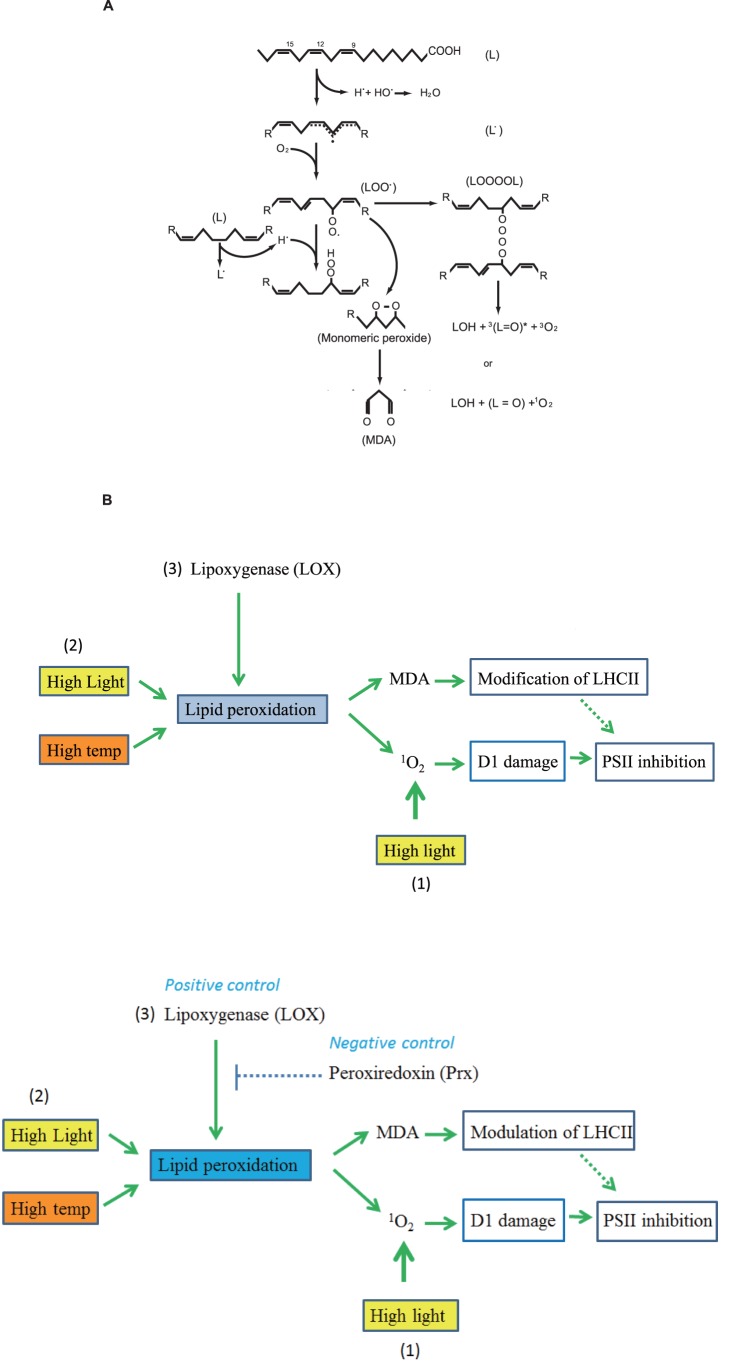
A schematic diagram of lipid peroxidation and a model showing a pathway of damage to the D1 protein and LHCII which is triggered by lipid peroxidation. (**A**) A detailed mechanism for lipid peroxidation and the related processes. (**B**) A diagram showing proposed pathways for the light-induced damage to the D1 protein and LHCII. (1) a well- known route showing the light-induced production of ^1^O_2_ at the reaction center of PSII and damage to the D1 protein. (2) a new pathway of damaging the D1 protein through lipid peroxidation and the subsequent formation of ^1^O_2_ and MDA suggested in the present study. (3) lipoxygenase-induced lipid peroxidation and damage to the D1 protein.

We explain the damage by the action of ROS produced through photochemical reaction at PSII and also through lipid peroxidation (Fig. 7). There are probably two causes for the damage. First, ^1^O_2_ produced after lipid peroxidation may damage the D1 protein and LHCII. As the concentration of LOO^•^ increases, it forms a tetroxide intermediate LOOOOL by the Russell-type reaction. The decomposition of tetroxide forms LOH and either triplet excited carbonyl ^3^(L = O)^*^ and molecular oxygen or ground state carbonyl (L = O) and ^1^O_2_ [20]. Singlet oxygen is a potential oxidant and may damage the D1 protein and LHCII, as has been demonstrated with the acceptor-side photoinhibition of PSII [3], [4]. Second, the products from decomposition of lipid hydroperoxides [20], [26], [43] may damage LHCII. The secondary products include reactive carbonyl compounds, of which the most abundant is MDA. MDA can be present as a free form, but it reacts readily with the lysine residues of proteins to form a variety of cross-linked products [26]. The modification of the proteins by the secondary products of lipid peroxidation may inevitably cause damage to the function of the proteins. For example, modification of an extrinsic PsbO protein of PSII by MDA in heat-stressed plants was reported [44]. Previously we showed formation of cross-linked products of the D1 protein with antenna chlorophyll-binding protein CP43 and the α-subunit of cyt *b*_559_ under light stress and heat stress [3], [4], [27], [28], [29], [45], [46]. The protein cross-linking was explained by direct reaction between the photodamaged D1 protein and the nearby proteins. However, our present results suggest an important role of lipids as a mediator of the cross-linking reaction. More recently, lipid peroxidation caused by the donor-side photoinhibition of PSII was reported with alkaline-treated Mn-depleted spinach PSII membranes [23]. These results are important because they suggest presence of possible modification of the D1 protein by lipid peroxidation under these conditions. By contrast, the present illumination conditions that we used here at 20–30°C correspond to that inducing the typical acceptor-side photoinhibition of PSII, and hence our results show that the acceptor-side photoinhibition of PSII causes lipid peroxidation, which subsequently gives damage to the proteins of PSII. Taking all these results into account, we suggest that there exists a linkage between light-induced lipid peroxidation, generation of ^1^O_2_ and MDA, and damage to the D1 protein and LHCII (Fig. 7). The effects of lipid peroxidation may be subsidiary in damaging PSII, but the effects are substantial, particularly in the thylakoids. Marked difference between the thylakoids and the PSII membranes in regard to the susceptibility to lipid peroxidation indicates that the bound lipids in and around PSII are relatively resistant to lipid peroxidation.

## Materials and Methods

### Preparation of the Thylakoids and PSII Membranes from Spinach

Fresh spinach leaves were purchased from a local market. Intact thylakoid membranes were prepared according to the previously described method [47]. We omitted 5 mM Na-ascorbate in the grinding medium to avoid the effects of the reductant on the lipid peroxidation in the samples. Thylakoids were suspended in a buffer solution containing 0.1 M sorbitol, 15 mM NaCl, 5 mM MgCl_2_, 30% (v/v) ethylene glycol and 50 mM Tricine-KOH (pH 7.5) (solution A), frozen in liquid nitrogen and stored at –30°C until use. For all the experiments, the thylakoid membranes were washed once and suspended with solution A without ethylene glycol (solution B). PSII membranes were prepared as described previously [21], frozen in liquid nitrogen and stored at –30°C until use. All procedures were carried out in darkness under green safe light. Chlorophyll was determined with 80% acetone extract using a Hitachi U-2000 spectrophotometer (Japan).

### Illumination of the Thylakoids and PSII Membranes

The thylakoids or PSII membranes (0.1 mg chlorophyll mL^–1^) were placed in 0.5 mL transparent plastic tubes and incubated under illumination with white light (light intensity, 100–1,000 µmol photons m^−2^ s^−1^) for the indicated periods of time in a thermostat water bath set at either 20°C or 30°C. The incubation was terminated by transferring the samples in the microtubes to an ice bath in the dark.

### Analysis of Lipid Peroxidation

Lipid peroxidation was measured with the TBARS assay or using a fluorescence probe developed to monitor lipid peroxide. For the TBARS assay, we used a NWLSS malondialdehyde assay kit (Northwest Life Science, USA). This assay is based on the reaction of MDA with TBA. The amount of MDA was estimated from the absorbance at 532 nm of the TBA–MDA complex in the thylakoids and PSII membranes using a Hitachi U-3900 spectrophotometer (Japan). Absorbance at 600 nm was subtracted from that at 532 nm to eliminate contribution of a nonspecific absorbance. The fluorometric assay of lipid peroxide was done according to Khorobrykh et al [23]. A fluorescence probe 2-(4-diphenylphosphanyl-phenyl)-9-(1-hexyl-heptyl)-anthra[1,1,9-def,6,5,10-d′e′f′]diisoquinoline-1,3,8,10-tetraone (Spy-LHP) was purchased from Dojindo, Japan. Spy-LHP was dissolved in ethanol at the concentration of 3 µM and incubated at 37°C for 15 min. Fluorescence was measured with a Hitachi fluorescence spectrophotometer F-2500. Excitation and emission wavelengths were 524 nm and 538 nm, respectively.

### Treatment of the Thylakoids and PSII Membranes with Lipoxygenase

LOX from soybean (*Glycine max*) was purchased from Sigma (USA). Unless otherwise stated, LOX solution (1 mg LOX mL^–1^) was added to the thylakoids or PSII membranes suspended in solution B, which contained 0.1 mg chlorophyll mL^−1^ in order to make 0.1–0.5 mg LOX mL^–1^. The suspensions were incubated at either 20°C or 30°C in the dark for 30 min.

### Preparation of Malondialdehyde and Treatment of the Thylakoids and PSII Membranes with Malondialdehyde

MDA was prepared by acid hydrolysis of 1,1,3,3-tetramethoxypropane at pH1 at 40°C for 2 h. After neutralization with NaOH, the concentration of MDA was determined from UV absorption at 267 nm using a molar extinction coefficient of 3.42×10^4^ M^−1^ cm^−1.^

[48]. MDA was added to the thylakoids or PSII membranes at final concentration of 100 or 500 µM and the whole suspensions were incubated at either 20°C or 30°C in the dark for 60 min.

### Sodium Dodecyl Sulfate/Urea-polyacrylamide Gel Electrophoresis and Western Blot Analysis

SDS/urea-polyacrylamide gel electrophoresis (PAGE) and Western blot analysis were carried out as previously described [47]. Concentrations of acrylamide in the stacking and resolving gels were 5% (w/v) and 12.5% (w/v), respectively. In each lane of the gel, the thylakoids or PSII membranes equivalent to 10 µg of chlorophyll was included. The antibody against the DE-loop of the D1 protein was used for detection of the cleavage and aggregation products of the D1 protein after exposure of the samples to excessive light or after incubation with LOX. A horseradish peroxidase-conjugated anti-rabbit antibody or an anti-hen antibody (Bio-Rad, Japan) was used as the secondary antibody. Antibodies against *Arabidopsis* light harvesting chlorophyll-protein subunits of PSII (Lhcb1-6) and *Arabidopsis* LOX were purchased from AgriSera (Sweden). An antibody against MDA was purchased from Nichiyu, Japan. Immuno-decorated bands were detected by fluorography with enhanced chemiluminescence (ECL) (Amersham, Japan).

### Determination of Protein Oxidation

Protein oxidation was measured according to the method previously described [21]. The carbonyl content of protein was measured using an Oxi-Blot protein oxidation detection kit (Chemicon International, USA). Protein samples (5 µg/lane) were derivatized with 2,4-dinitrophenyl hydrazine, assayed by 12.5% acrylamide/SDS-PAGE, and electroblotted to poly(vinylidene difluoride) membranes (Millipore, Japan). The derivatized proteins were sequentially reacted with rabbit anti-DNP and horseradish peroxidase-conjugated goat anti-rabbit IgG antibodies, and visualized by ECL.

### Measurement of Photosystem II Activity

Chlorophyll fluorescence Fv/Fm was monitored using a Mini-PAM (Walz, Germany) at 20°C. The thylakoids or PSII membranes equivalent to 0.1 mg Chl ml^−1^ were suspended in solution B. For the assay of the effects of LOX on the PSII activity, the membrane samples were incubated in the reaction mixture containing 0.4 M sucrose, 50 mM Hepes-KOH (pH7.0), 10 mM NaCl, 5 mM MgCl_2_. The samples were dark-adapted for at least 30 min on ice.

### Electron Paramagnetic Resonance Spin-trapping Spectroscopy

Hydrophilic spin trap compound TMP (Sigma) was used for ^1^O_2_ detection. Oxidation of diamagnetic TMPD by ^1^O_2_ yields a paramagnetic TEMPONE EPR signal [49]. Before use, TMPD was purified twice by vacuum distillation to eliminate the TEMPONE EPR signal because of impurities in the spin trap. Prior to strong light treatment, 50 mM TMPD was added to PSII membranes (500 µg chlorophyll mL^–1^) in 40 mM Mes (pH 6.5). Strong light treatment was performed under a continuous gentle stirring with a continuous white light (100–1,000 µmol photons m^−2^ s^−1^) using a halogen lamp with a light guide (Schott KL 1500, Schott AG, Mainz, Germany). For LOX treatment, PSII membranes (500 µg chlorophyll mL^–1^) in 25 mM phosphate buffer (pH 7) were incubated with LOX (0.1–0.5 mg mL^–1^) at 30°C in the presence of 50 mM TMPD. The incubation was performed in complete darkness under continuous gentle stirring. After light or LOX treatments, the sample was centrifuged at 5,000 × g for 3 min to separate TEMPONE from PSII membranes. The separation of two phases was performed to avoid the reduction of TEMPONE by a non-specific reducing component in the PSII membranes. After centrifugation, the upper phase (TEMPONE) was transferred into the glass capillary tube (Blaubrand® intraMARK, Brand, Germany), whereas the lower phase (PSII membranes) was discarded. To assess the number of spin trapped in the polar phase, TEMPONE EPR spectra was measured using ultra-pure TEMPONE (Alexis Biochemicals, Lausen, Switzerland). The glass capillary tubes were kept in liquid nitrogen until use. Prior to the measurements, the capillary tube was taken away from the liquid nitrogen and EPR spin-trapping spectra were collected at room temperature using an EPR spectrometer MiniScope MS200 (Magnettech GmbH, Germany). The EPR spectrum was obtained as a first derivate of the EPR absorption signal. EPR conditions were as follows: microwave power, 10 mW; modulation amplitude, 1 G; modulation frequency, 100 kHz; sweep width, 100 G; and scan rate, 1.62 G s^–1^.

## Supporting Information

Figure S1 **Western blot analysis of spinach thylakoids with the antibody against LOX. (A**) Coomassie blue-staining of the proteins in spinach thylakoids after SDS-PAGE examination of the presence of endogenous LOX in the thylakoids. Where indicated as “+ LOX”, soybean LOX was added to the thylakoids at a concentration of 0.1 mg mL^−1^ as a positive control for the presence of LOX. Samples were loaded on the basis of the same chlorophyll (2.5 µg of chlorophyll). Molecular markers are shown at the left side of the gel. Several proteins including the externally added LOX are also indicated at the right hand side of the gel. (**B**) Western blot analysis with an antibody against soybean LOX. The position of LOX and the relative molecular mass of LOX (96 kDa) are shown at the left-hand side and right-hand side of the gel, respectively.
(EPS)

Figure S2 **Effects of light stress on LHCII of the thylakoids and PSII membranes. (A)** SDS/urea-PAGE gel showing the Coomassie-stained proteins of the thylakoids and PSII membranes after illumination with strong light (light intensity: 1,000 µmol photons m^−2^ s^−1^) for 30 and 60 min. The molecular markers and the position of LHCII are indicated at the left side and right side of the gel, respectively. At the bottom, the band of Lhcb1 assayed by Western blotting is shown. (**B)** A fluorogram of Western blot analysis showing the change in the amounts of Lhcb1-6, the D1 protein, CP43 and CP47 after strong illumination of the thylakoids (left). The light intensity was the same as that in *A*. Appearance of the aggregates of Lhcb1 is shown with the Western blot analysis during 60 min illumination (right). The molecular markers are shown at the right side of the fluorogram. (**C)** A fluorogram of Western blot analysis showing the aggregates of the D1 protein, Lhcb1 and CP43 formed by strong illumination of the thylakoids for 60 min (right). Dark control is also shown (left). The arrow indicates the aggregates commonly observed in the D1 protein and Lhcb1 and therefore seem to be formed by cross-linking between the D1 protein and Lhcb1.
(EPS)

Figure S3 **Effects of malondialdehyde on the PSII activity of the thylakoids. (A**) The absorption spectrum of the MDA formed by acid hydrolysis of 1,1,3,3-tetramethoxypropane with HCl carried out at pH 1 at 40°C for 2 h. As MDA was neutralized by the addition of NaOH, the enolate anion having absorption maximum of 267 nm is the predominant form. (**B**) The effects of MDA on the chlorophyll fluorescence Fv/Fm of the thylakoids. The samples were incubated with MDA of given concentrations at either 20°C or 30°C for 30 min. On the ordinate, the relative value of Fv/Fm is shown. The data are the means of three measurements±S. D.
(EPS)
