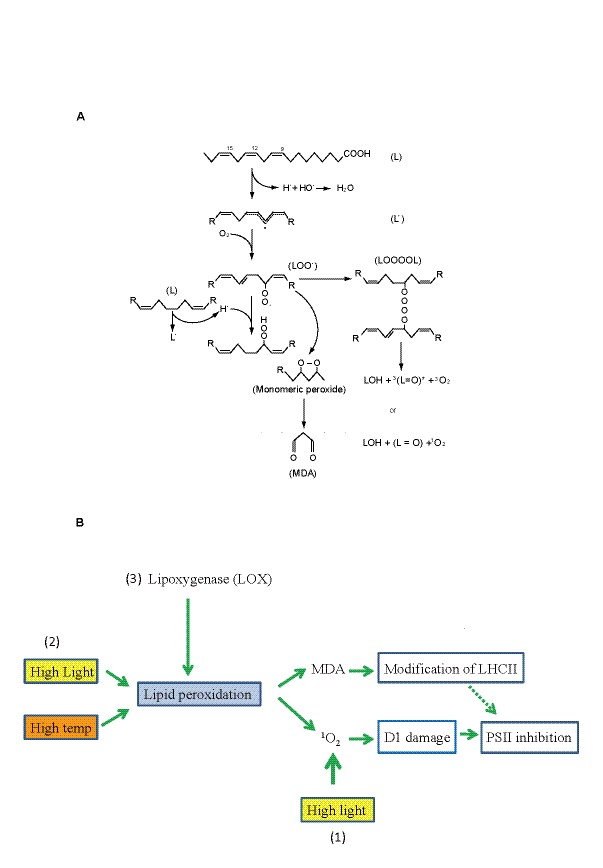# Correction: Quality Control of Photosystem II: Lipid Peroxidation Accelerates Photoinhibition under Excessive Illumination

**DOI:** 10.1371/annotation/8b0f8019-e4ba-4c35-9575-1a1313ae7b41

**Published:** 2013-05-28

**Authors:** Tiffanie Chan, Yurika Shimizu, Pavel Pospíšil, Nobuyoshi Nijo, Anna Fujiwara, Yoshito Taninaka, Tomomi Ishikawa, Haruka Hori, Daisuke Nanba, Aya Imai, Noriko Morita, Miho Yoshioka-Nishimura, Yohei Izumi, Yoko Yamamoto, Hideki Kobayashi, Naoki Mizusawa, Hajime Wada, Yasusi Yamamoto

There was an error in Figure 7.

The correct version of Figure 7 is available here: 

**Figure:**